# SOCIAL CONNECTIVITY IS ASSOCIATED WITH INCREASED PERFORMANCE ON MEDIAL TEMPORAL LOBE FUNCTION IN OLDER AFRICAN AMERICANS

**DOI:** 10.1093/geroni/igac059.1768

**Published:** 2022-12-20

**Authors:** Genesis Tan, Zuzanna Osiecka, Bernadette Fausto, Darlingtina Esiaka, Mark Gluck

**Affiliations:** Rutgers University - Newark, Newark, New Jersey, United States; Rutgers University - Newark, Newark, New Jersey, United States; Rutgers University - Newark, Newark, New Jersey, United States; Robert Wood Johnson Medical School, Rutgers University, Newark, New Jersey, United States; Rutgers University - Newark, Newark, New Jersey, United States

## Abstract

Research links social connectivity to cognitive function in older adults. However, many of the studies relating social connectivity to cognitive function focused on global cognition—orientation/attention, memory, verbal fluency, language, and visuospatial ability domains. In this study, we aimed to assess the association of social connectivity with generalization, a domain of cognition which relies on hippocampal function, in a population of older African Americans residing in the Greater Newark area. Specifically, we examined the impact of social connectivity on generalization using sensitive measures that tap into medial temporal lobe (MTL) function. Participants (N = 74; M = 73.84) from an ongoing study, Pathways to Healthy Aging in African Americans—a Rutgers University-Newark community partnership fostered over 16 years of community engagement, health education, and public service—responded to measures of cognitive function, social network, social engagement, mental health, and demographic details. Also, they completed a task-based functional Magnetic Resonance Imaging. Results showed that marital status was negatively associated with MTL function, with those that are either single, divorced, or separated outperforming those that are married. Similarly, depressive symptoms had a negative association with MTL function. Further, the linear combination of social network variables and covariates significantly predicted MTL function. Our findings illuminate the benefits of social connectivity and resources on cognitive skills, and amplify the need to study the brain in the social context.

